# Efficacy, Satisfaction, and Safety of Low-Intensity, Large-Area Shock Wave Therapy With Dual-Probe Energy Delivery for Erectile Dysfunction

**DOI:** 10.7759/cureus.83389

**Published:** 2025-05-03

**Authors:** Jun Kato, Masato Shirai, Keisuke Ishikawa, Akira Tsujimura

**Affiliations:** 1 Department of Urology, East Ekimae Clinic Shinbashi, Tokyo, JPN; 2 Department of Urology, Juntendo University Urayasu Hospital, Urayasu, JPN

**Keywords:** erectile dysfunction, erection hardness score, satisfaction, sexual health inventory for men, shockwave treatment

## Abstract

Objective: We evaluated the efficacy and safety of a new low-intensity extracorporeal shockwave therapy generator, the Morenova system (Direx Systems GmbH, Wiesbaden, Germany), that offers fewer treatments and shorter treatment times through dual-probe energy delivery for patients with erectile dysfunction (ED). We additionally examined whether aging affects treatment efficacy and whether there are independent factors that predict treatment efficacy. To evaluate efficacy from the viewpoint of patient satisfaction, free comments written by the patients were analyzed by artificial intelligence (AI) to clarify the percentage of responses judged to be “positive.”

Methods: This study included 64 male patients (56.5 ± 1.5 years) with ED who had undergone treatment with the Morenova. The treatment involves delivering a total of 1,800 shock waves (900 from each of the two applicators) with an energy density of 1.8 mJ/mm^2^ to the sides of the penis and the perineum. Each patient underwent five sessions conducted two to three times per week. Efficacy was evaluated by two specific questionnaires (Sexual Health Inventory for Men, SHIM, and Erection Hardness Score), and adverse events were confirmed by direct questioning of the patients. The degree of improvement was evaluated for five age groups of patients categorized in their 30s, 40s, 50s, 60s, and 70s and older. The patients’ free-form feedback on their impressions and opinions of the treatment was also evaluated by AI.

Results: We found significant improvement between pretreatment and one-month posttreatment score of the SHIM (from 11.5 ± 0.5 to 16.6 ± 0.7; p < 0.01) and Erection Hardness Score (from 1.94 ± 0.09 to 2.83 ± 0.10; p < 0.01), respectively. Significant improvement between pretreatment and three-month posttreatment scores from the two questionnaires was also found (from 11.5 ± 0.5 to 16.6 ± 0.7; p < 0.01 and from 1.94 ± 0.09 to 2.66 ± 0.13; p < 0.01, respectively). Morenova treatment caused no adverse events. The degree of improvement was not affected by the patients’ age. Only pretreatment Erection Hardness Score was found to be an independent influencing factor for the efficacy of the treatment. Analysis by AI of patients’ free-form feedback showed that 70% of the opinions were positive.

Conclusions: We concluded that treatment with the Morenova system is an effective and safe option for ED, which should be tried in all age groups, as approximately 70% of patients reported satisfaction with the treatment. The patients appeared to be satisfied with treatment by Morenova because of fewer clinic visits and shorter treatment times, coupled with a good response.

## Introduction

Recently, the young generation has focused increased attention on erectile dysfunction (ED) because of problems with a reduction in the birthrate. The theory that ED may show the possibility of a rise in concomitant cardiovascular events caused by atherosclerosis has attracted a lot of attention in the older generation. The prevalence of men with ED worldwide was estimated at over 152 million in 1995 and is predicted to increase up to approximately 322 million in 2025 [1]. The Japanese Society for Sexual Medicine (JSSM) conducted a nationwide survey on the actual status of sexual function targeting men aged ≥20 years in 2023 [2]. The results revealed the prevalence of ED to be 30.9%, with approximately 14 million men affected, as assessed by the Erectile Hardness Score (EHS) [3]. Since the first official national survey in Japan was conducted in 1998, which reported 11.3 million men with moderate or complete ED, it was calculated that the number of patients with ED has increased by less than three million in Japan over the past 25 years [2]. According to the guidelines of the American Urological Association [4], the European Association of Urology [5], and the JSSM/Japanese Urological Association [6], phosphodiesterase type 5 inhibitors (PDE5Is) have been the first-line treatment for ED. If PDE5Is are ineffective or contraindicated, intracavernosal injection of a vasoactive agent such as prostaglandin E1 and vacuum erectile devices are commonly recommended as a second option. However, prostaglandin E1 for intracavernosal injection is only approved for examination and cannot be used for treatment in Japan. Therefore, the only remaining option is the one vacuum erectile device that is approved by the Pharmaceuticals and Medical Devices Agency and was launched in 2022. Recently, we reported the efficacy of that vacuum erectile device and showed that of the 16 patients who could not insert during sexual intercourse, 10 (62.5%) were able to insert after the treatment [7]. However, there were a few patients who were not satisfied because they could not handle the device well or because it disturbed the atmosphere of sexual activity. It is also true that some people did not want on-demand treatment with vacuum erectile devices but rather fundamental improvement of penile blood flow and thus an increase in their erectile strength. In the current situation, another treatment option that has recently gained attention is low-intensity extracorporeal shockwave therapy (LiESWT). However, this treatment is not covered by national health insurance in Japan or in other foreign countries. However, its use has been increasing in Japan, especially among patients with vascular ED. Presently, an electromagnetic lithotripter, the Renova (Direx Systems GmbH, Wiesbaden, Germany), which requires only four weekly sessions of 3,600 shocks (energy density = 0.09 mJ/mm^2^, frequency = 5 Hz), has been implemented in over 70 countries and boasts over 100,000 treatment cases. The therapy itself is relatively painless, takes about 20 minutes, and has not been associated with any side effects, making it a promising new strategy in ED treatment. We already reported that all treatments were safe in 484 Japanese patients, and the effective rate based on patient satisfaction was 71.4% [8].

In the present study, we evaluated the efficacy and adverse events of treatment by the next LiESWT generator of the Renova product family, the Morenova (Direx Systems GmbH, Wiesbaden, Germany), which offers dual-probe energy delivery for efficient tissue coverage of a larger surface area in less time. The Morenova is basically a compact version of the Renova, and while the fundamental principle remains the same, it delivers low-intensity shock waves to the penis and perineum. We also investigated whether efficacy varied with age and analyzed factors defining the degree of improvement of erection. Finally, we evaluated our patients’ satisfaction based on responses obtained from their free comments.

## Materials and methods

This retrospective study included 64 male patients who had undergone LiESWT with Morenova from May 2022 to March 2024. All patients complained of some level of problem with erections. Patients with a history of radical prostatectomy or extensive pelvic surgery, patients with abnormal bleeding tendency or coagulopathy as indicated by an International Normalized Ratio of 3 or higher, hemophiliacs, and those who had undergone pelvic region surgery or radiation therapy or cancer treatment within the past 12 months were excluded from treatment, as were patients with psychiatric disorders, Peyronie’s disease, or penile implants.

Age, sexual symptoms evaluated by two specific questionnaires, including the Sexual Health Inventory for Men (SHIM) [9] and the EHS, and the duration of ED were assessed. Additionally, patients were asked to provide free-form feedback on their impressions and opinions of the therapy one month after the end of treatment. Metabolic comorbidities such as hypertension, diabetes, and hyperlipidemia were also assessed. Patients were asked if they had taken PDE5Is in the past, and those patients were also asked to indicate their erectile status when not taking the drug. Not all patients who were taking PDE5Is were satisfied with their efficacy.

**Figure 1:**
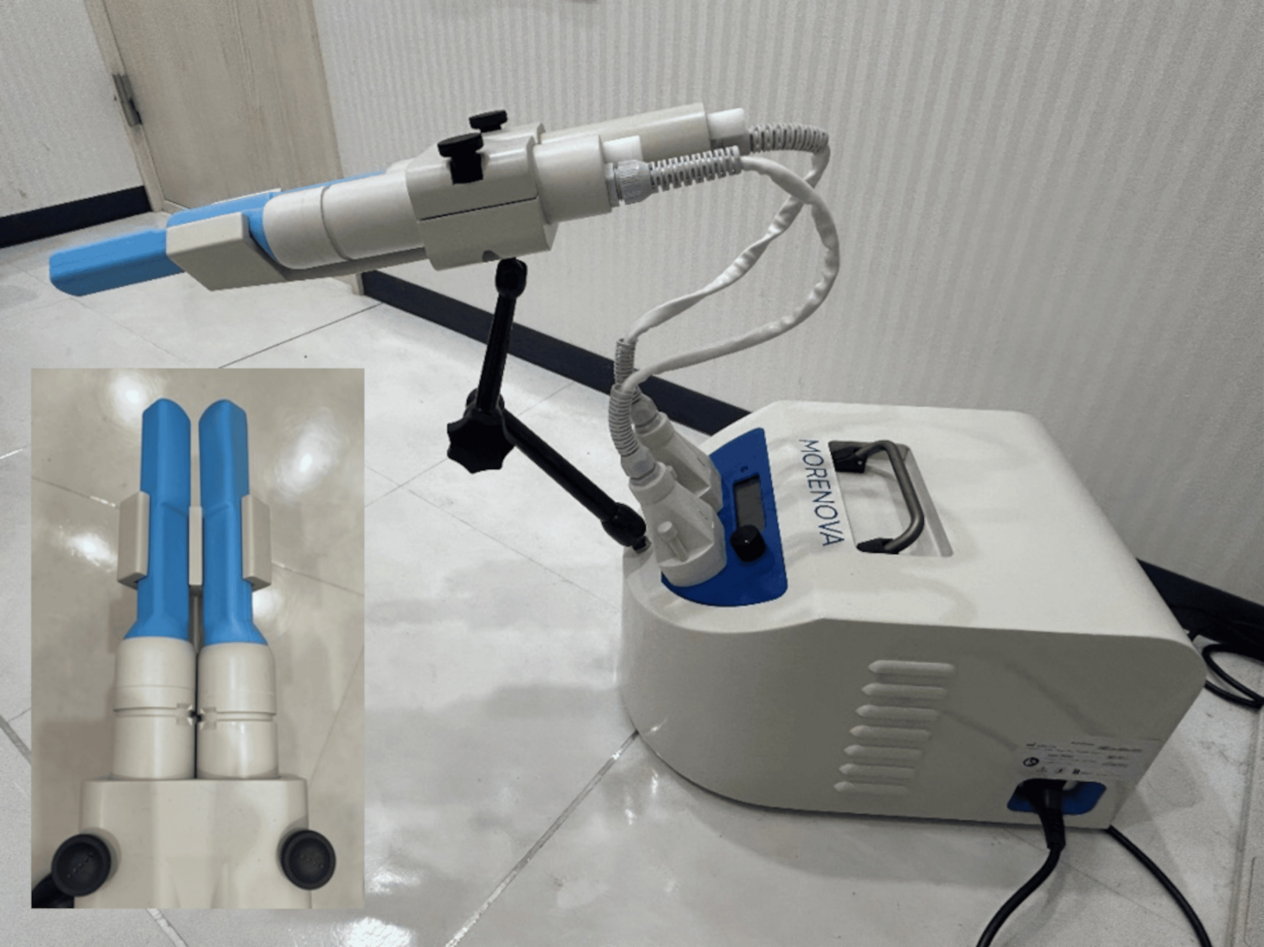
Morenova system It uses an electromagnetic linear generator with dual-probe energy delivery at an energy density of 0.09 mJ/mm^2^

Morenova utilizes an electromagnetic linear generator with dual-probe energy delivery, achieving an energy density of 0.09 mJ/mm², similar to the Renova, as illustrated in Figure 1. Morenova is a compact lithotripter with a width of 290 mm, depth of 400 mm, height of 230 mm, and weight of 9 kg. Patients can be treated in a sitting position with legs slightly open.

The treatment involves delivering a total of 1,800 shock waves (900 from each of the two applicators) to the sides of the penis and the perineum. An energy density of 0.09 mJ/mm² is the standard energy for ED treatment with extracorporeal shock waves. With the Morenova, we aim to deliver this energy to the targeted organs. The important criteria in low-intensity shockwave therapy are as follows: 1) the target energy density (0.09 mJ/mm²) is achieved to maximize the angiogenetic response of the targeted tissue, 2) the area/volume of tissue receiving the energy, and 3) the number of repetitions (i.e., number of shocks administered).

As there is still no standard protocol for frequency of treatment, we followed the protocol recommended by Direx Systems GmbH, which entails five sessions conducted two to three times per week. Each session of the treatment took about 20 minutes, including preparation.

To determine efficacy, the SHIM score and EHS were compared before and one and three months after treatment. The degree of change in SHIM (ΔSHIM) and EHS (ΔEHS) was evaluated for five groups of patients categorized in the age groups of 30s, 40s, 50s, 60s, and 70s and older. Because the SHIM scores are very low in the absence of sexual intercourse, factors related to ΔEHS, which can objectively assess erectile hardness rather than SHIM, were evaluated by multivariate analysis. Finally, free comments including information on adverse events were sorted into positive, neutral, and negative opinions by artificial intelligence (AI: ChatGPT, OpenAI, San Francisco, CA). A positive opinion was defined by individuals who reported feeling the effects of the treatment, expressed satisfaction, mentioned improvements in their condition, believed the treatment was effective, and showed high interest in receiving additional treatments and undergoing repeated sessions. A neutral opinion was defined by individuals noting that there was some effect, but they were not fully satisfied, and they felt somewhat disappointed or experienced no particular change in their condition. A negative opinion was defined by individuals expressing that they felt no effect from the treatment and were disappointed, the mention of financial concerns making it difficult for them to pursue additional treatments, the continued struggle to maintain erections, and the finding that the treatment did not fully address their issues.

This study was conducted in accordance with the Declaration of Helsinki and Good Clinical Practice guidelines, and written informed consent was obtained from all subjects. The procedures were approved by the Regional Ethics Committee of Juntendo University, Japan (approval number: E23-0445-U01).

Statistical analysis

Data are basically presented as the mean ± standard error. Changes in the symptom scores were evaluated by a paired t-test. To evaluate changes in ΔSHIM and ΔEHS, a comparison was made between the five age-range groups by the Kruskal-Wallis test. Independent influencing factors for ΔEHS were analyzed by multiple regression analysis. Statistical significance was set at p < 0.05. Statistical analyses were performed with IBM SPSS Statistics for Windows, version 24.0 (IBM Corp., Armonk, NY).

## Results

The characteristics of the 64 patients who underwent LiESWT with Morenova are shown in Table 1.

**Table 1:** Clinical characteristics of 64 patients with ED treated by Morenova as low-intensity extracorporeal shock wave therapy SHIM: Sexual Health Inventory for Men; EHS: Erection Hardness Score; ED: erectile dysfunction; PDE5I: phosphodiesterase type 5 inhibitor

Characteristic	Value (n = 64)
Age (years)	56.5 ± 1.5
SHIM	11.5 ± 0.5
EHS	1.94 ± 0.09
Duration of ED (months)	3.6 ± 0.4
Hypertension	9 (14.1%)
Diabetes	4 (6.3%)
Hyperlipidemia	6 (9.4%)
History of taking PDE5I	60 (94.5%)

All patients completed the treatment sessions, and no adverse events such as pain and skin damage to the penis were noted. Patient age was 56.5 ± 1.5 years, and the SHIM score and EHS were 11.5 ± 0.5 and 1.9 ± 0.1, respectively. Thus, the average status of erectile function of our patients showed moderate ED by the SHIM and less than grade 2 (penis is hard, but not hard enough for penetration) by the EHS. Duration of ED was 3.6 ± 0.4 months. The rates of metabolic factors associated with hypertension, diabetes, and hyperlipidemia were 14.1%, 6.3%, and 9.5%, respectively. Sixty patients (94.5%) had taken PDE5Is in the past.

**Figure 2:**
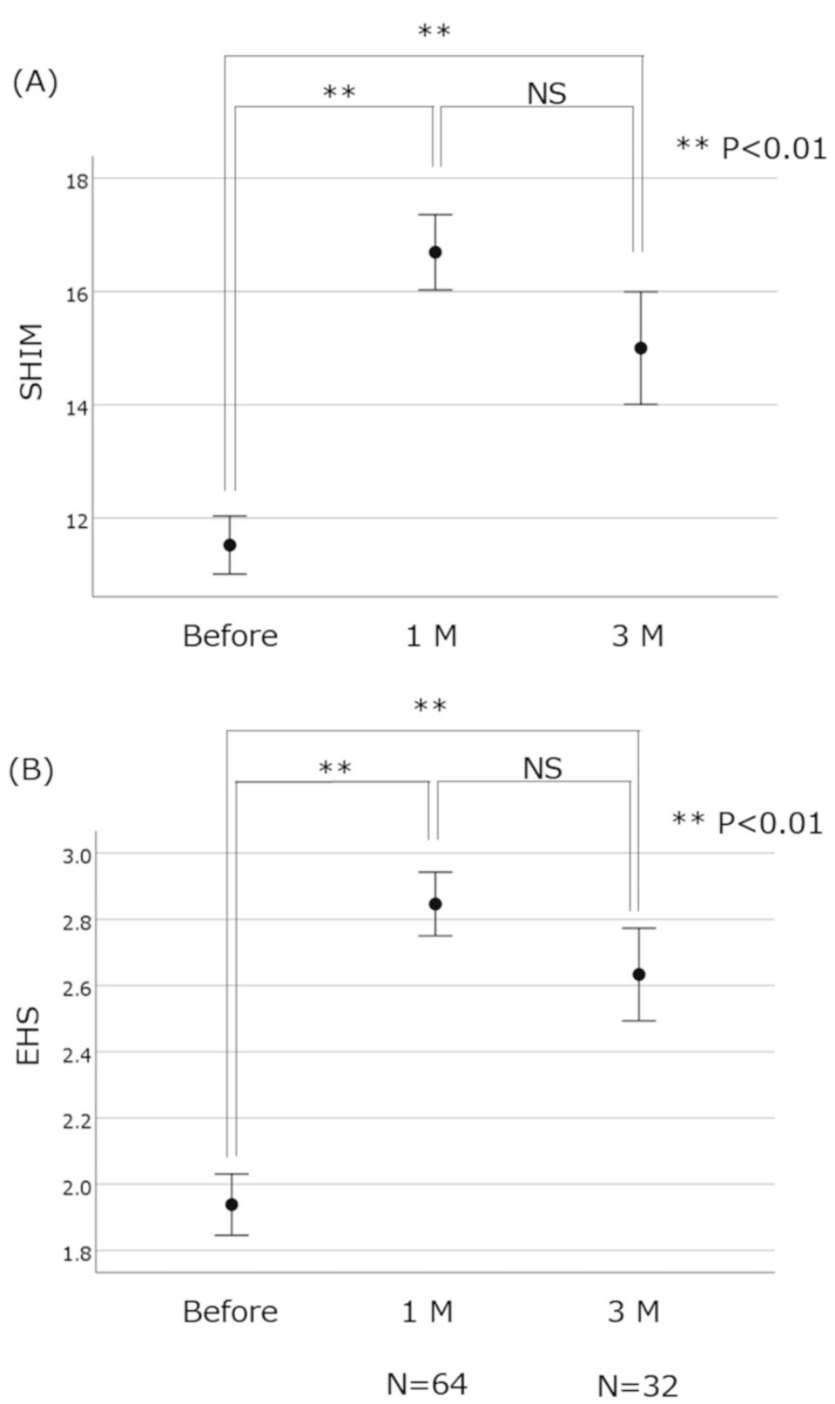
Efficacy between pretreatment and one-month posttreatment shown by (A) SHIM score and (B) EHS Significant improvement between pretreatment and one-month posttreatment was shown by the (A) SHIM score (from 11.5 ± 0.5 to 16.6 ± 0.7; p < 0.01) and (B) EHS (from 1.94 ± 0.09 to 2.83 ± 0.10; p < 0.01). Improvement between pretreatment and three-month posttreatment was also shown by the (A) SHIM score (from 11.5 ± 0.5 to 15.0 ± 0.9; p < 0.01) and (B) EHS (from 1.94 ± 0.09 to 2.66 ± 0.13; p < 0.01) SHIM: Sexual Health Inventory for Men; EHS: Erection Hardness Score; NS: not significant

A significant improvement between pretreatment and one-month posttreatment was shown by the SHIM score (from 11.5 ± 0.5 to 16.6 ± 0.7; p < 0.01) and EHS (from 1.94 ± 0.09 to 2.83 ± 0.10; p < 0.01), respectively (Figures 2A, 2B).

**Figure 3:**
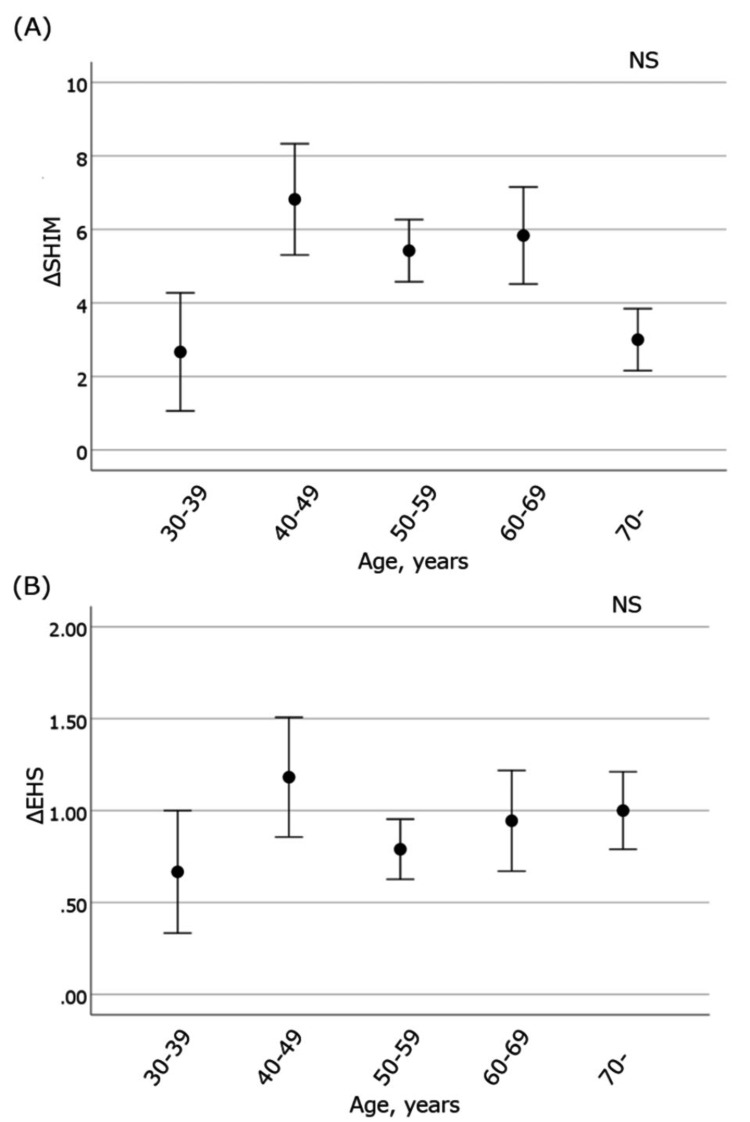
(A) ΔSHIM and (B) ΔEHS values between the five age-range groups spanning from the 30s to the 70s and older ΔSHIM and ΔEHS were not significant findings across the five age groups SHIM: Sexual Health Inventory for Men; EHS: Erection Hardness Score; NS: not significant

An improvement between pretreatment and three-month posttreatment was also shown by the SHIM score (from 11.5 ± 0.5 to 15.0 ± 0.9; p < 0.01) and EHS (from 1.94 ± 0.09 to 2.66 ± 0.13; p < 0.01), respectively (Figures 2A, 2B), although only 32 patients could be evaluated at three months after treatment. The change between one and three months did not differ significantly for either score. ΔSHIM and ΔEHS values between the five age range groups are shown in Figures 3A, 3B.

ΔSHIM and ΔEHS findings were not significant in the age ratings. Furthermore, only pretreatment EHS was found to be an independent influencing factor for ΔEHS in the multiple regression analysis (Table 2).

**Table 2:** Independent factors associated with the improvement of EHS in patients treated by Morenova at one month after treatment CI: confidence interval; EHS: Erection Hardness Score; ED: erectile dysfunction

Characteristic	Nonstandardizing coefficient	Standardizing coefficient	p	95% CI	
				Lower	Upper
Constant	1.796	-	0.032	0.156	3.436
Age	0.008	0.103	0.507	-0.016	0.032
EHS	-0.744	-0.614	0.000	-0.995	-0.494
Duration of ED	0.045	0.155	0.287	-0.038	0.128
Metabolic factor	-0.292	-0.106	0.355	-0.918	0.335

**Figure 4:**
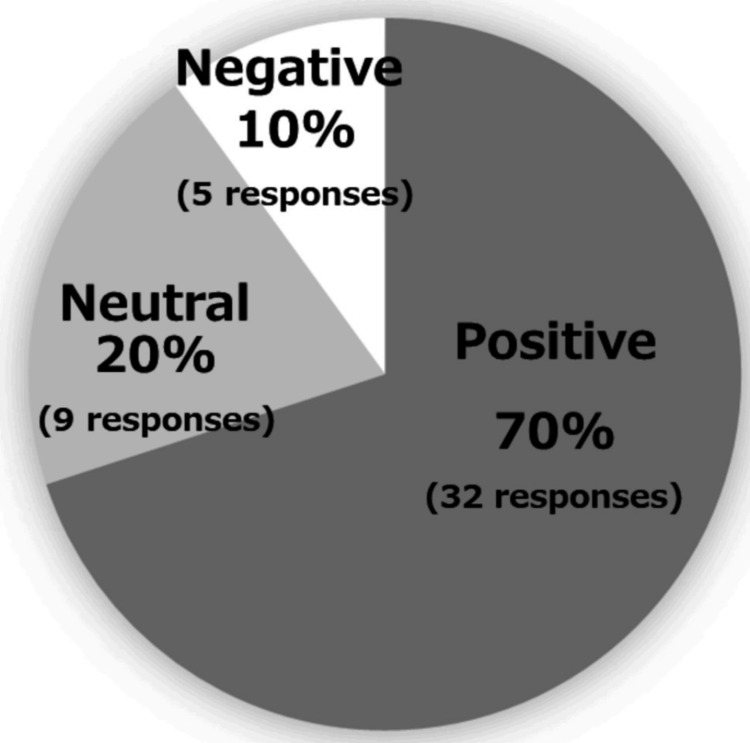
Satisfaction by the artificial intelligence analysis of the responses from our participants, which showed that 70% provided positive feedback

Figure 4 displays an AI analysis of the responses from our participants, which showed that 70% provided positive feedback (see the Appendix).

## Discussion

A United Nations study has predicted that patients with ED will increase from 152 million to approximately 322 million worldwide between 1995 and 2025 [1]. Additionally, the number of Japanese patients with ED has increased from 11.3 to 14 million over the past 25 years [2]. Furthermore, it is well known that many patients do not recover their erectile function with PDE5Is and desire another option for the treatment of their ED. As a result, LiESWT has gained increased attention in the field of sexual medicine in Japan.

Extracorporeal shock waves (ESWs) are waves of pressure transmitted above the speed of sound, and ESWs of different energy densities have different functions in clinical applications [10]. High-energy-density ESWs are often used in the field of urology to treat uroliths because of their emphasis on mechanical damage properties. Medium-energy-density ESWs have anti-inflammatory properties in the orthopedic field and are often used for surgical procedures such as bursitis and nonfracture treatments. Low-energy-density ESWs are expected to promote angiogenesis and improve blood supply and have recently been used in the treatment of cardiovascular disease [11]. A well-known important mechanism of ED is damage or impairment of vascular endothelial function [12,13]. LiESWT has been used to treat ED because it stimulates the expression of angiogenesis-related factors such as vascular endothelial growth factor, promotes vessel regeneration, and restores endothelial function [14]. The first study of the treatment of ED by LiESWT was reported in 2010 [15]. Since then, LiESWT has come into wide use in the clinical treatment of vascular ED [16], and numerous clinical studies on ED treatment with LiESWT have been reported. As scientific evidence, a systematic review and meta-analysis of 16 randomized controlled trials involving 1,064 patients was recently reported [17]. It found that 15 studies using the International Index of Erectile Function (IIEF) reported significantly improved scores after treatment compared to placebo, and in eight studies using the EHS, the score improved from <2 (sexually active, unable to insert) to 3 or more (able to insert), and patients receiving LiESWT were also significantly improved over those receiving placebo treatment. This systematic review and meta-analysis also reported that treatment with an energy density of 0.09 mJ/mm^2^ and 1,500-2,000 rounds is appropriate for improving IIEF and that patients with moderate ED are likely to benefit from the treatment. Recently, an open-label single-arm prospective study reported that 35 of the 52 patients (67.3%) with EHS <2 could achieve an erection hard enough for intercourse (EHS >3) after LiESWT treatment with PDE5i use [18].

As confidence in the effectiveness of LiESWT has increased, attention has focused on how to make the treatment less time-consuming. In other words, fewer treatments and shorter treatment times have become desirable. In this context, we already reported our experience with the ED1000, which requires 12 treatments, and Renova, which requires only four treatments (four weeks), and the efficacy was almost the same (70%) in both generators. However, it was apparent that patients preferred treatment with the Renova because of fewer clinic visits and shorter treatment times. Although the number of treatment sessions with Morenova in the present study was five, multiple treatments were given over one week, so the treatment period was completed within two to three weeks, which tended to be more preferred by the patients. However, the efficacy of Morenova has been reported in only one study. Treatment with Morenova at 0.09 mJ/mm^2^ was administered to 13 patients (mean age, 48.3 years) at 600 pulses per session every other day for two weeks for a total of six sessions (total, 3,600 rounds), and after treatment, the IIEF was significantly improved at three and six months compared to that before treatment [19]. However, eight patients (mean age, 60.3 years) treated with 720 pulses each for five consecutive days (total, 3,600 rounds) did not show a statistically significant improvement in IIEF after treatment [19]. Although the data suggest that a certain treatment interval may be necessary, the number of cases is too small, and there are differences in patient backgrounds, including age, that make a scientific judgment difficult. However, taking these factors into account, we followed a protocol of two to three treatments per week for a total of five sessions.

In the present study, we clearly showed that SHIM and EHS were significantly improved by treatment with Morenova. Furthermore, the treatment effects last at least three months, although the usefulness after that period should be discounted because both the SHIM and EHS at three months after the treatment were lower than those at one month after treatment, and only 32 patients (50%) were evaluable through three months. However, the evaluation of free comments from patients by AI showed that exactly 70% of them responded satisfactorily (positively) to the treatment. This is similar to the patient satisfaction rate of 71% in our previous clinical study with Renova and is considered a satisfactory result. The multivariate analysis reveals that patients with lower pretreatment EHS, i.e., more severe ED, are more likely to benefit from Morenova. This is an important finding for both patients and physicians, as the results indicate that patients do not hesitate to receive treatment due to the severity of their ED. Furthermore, in the present study, ΔSHIM and ΔEHS were compared in the five age-range groups and showed no difference in the amount of change depending on age. In our previous study with Renova, ΔEHS decreased in patients over 70 years of age, and age was an independent factor for a decrease in ΔEHS. In the present study, differences by age were not statistically significant, but this may be due to differences in the number of cases studied and to differences in patient backgrounds, such as the proportion of patients with diabetes mellitus. This point will require reevaluation after the number of cases increases. This study demonstrates the usefulness of Morenova for ED in Japanese patients. However, it should be emphasized that the guidelines of the American Urological Association and the European Association of Urology do not yet describe LiESWT as a highly recommended treatment. Rather, they describe it as an investigational treatment, and caution must be exercised in evaluating its outcome. Further accumulation and analysis of data will be required.

The present study has several limitations. First, the number of cases is small for the evaluation of efficacy. Second, the three-month observation period was short, and the number of patients who could be followed continuously through the third month was reduced to half. However, there were no significant differences between patients who were able to follow up at three months and those who were not able to, in terms of age; duration of ED, EHS, and SHIM before treatment; presence of metabolic factors; or history of PDE5Is use. Third, the small number of patients who had never taken PDE5I made it impossible to assess whether taking a PDE5I would affect treatment efficacy. Fourth, because this is a chart-based, backward-looking study, there is no comparison with a control group that does not emit shock waves, so a placebo effect cannot be ruled out. Finally, because of the treatment protocol of multiple treatments per week for a total of five treatments, the treatment intervals were not consistent across individuals.

## Conclusions

Despite several limitations, we believe that treatment with Morenova is an option that should be tried in all age groups, as it has been shown to be satisfactory in approximately 70% of patients treated over a short period of time and has proven useful, as indicated in evaluations using the SHIM and EHS. The patients in the present study appeared to prefer treatment with Morenova because of fewer clinic visits and shorter treatment times, coupled with a good response. However, long-term clinical trials and comparative studies with other ED treatment methods are crucial and are expected to clarify the positioning of Morenova in the treatment of ED. In addition, it is expected that, in the future, an even more compact generator could be fabricated that could be taken home and used by patients themselves. Patients would likely prefer this method because they may not need to go to a medical institution for therapy, and Morenova may be one device that could allow this form of treatment.

## Appendices

Artificial intelligence analysis of the responses from our participants

1. I think there has been some improvement.

2. Sometimes when I drink alcohol, I have a lack of hardness, but in normal times, I have no problems at all and am very happy with the results.

3. I thought it was an effective method.

4. I think the effect was noticeable two to three weeks after the treatment.

5. Compared to before the treatment, I can’t say that I have made a significant improvement yet, so I would like to see the progress from now on.

6. I could feel a definite change.

7. After a while, I would like to have another treatment as “insurance.”

8. I feel that my thickness has increased a little.

9. I feel that my semen has increased. Unfortunately, I have to use it in combination with Viagra to feel the effect.

10. I would like to have a complete treatment instead of drugs.

11. I would like to have a complete treatment, but it is difficult for me to have a treatment once a week for financial reasons.

12. Erection after the treatment is sure!

13. It was effective.

14. I think there was some effect, but not as much as I expected.

15. I feel like my whole body is better than before the treatment. Recently, I have been experiencing a lot of things that affect my autonomic nervous system, so that may have an effect as well.

16. There has been no visible effect.

17. There is also concern about a decrease in my ability to maintain an erection due to other factors.

18. Morning rutting has occurred often, but the response to sexual stimulation is still sluggish.

19. Erection hardness has increased, but would like to see more persistence. It's only been about a month, so I don't really feel that much.

20. Thank you for your help. I am satisfied with the results, but would like to have about one more Morenova treatment again.

21. Thanks to you, my stiffness has recovered considerably, but I am a little prematurely ejaculated, so I will do my best to improve it.

22. Thank you very much.

23. Thank you very much for your help.

24. I look forward to working with you again.

25. The effect was in waves, and I can't say that it was back to normal.

26. I have difficulty maintaining an erection.

27. I will see how it goes. I had an erection (index 3) before the treatment, but it became harder and erect (index 6) during masturbation from the third week after the treatment.

28. At one month after the treatment, I still have not achieved a satisfactory erection, but I thought Morenova was effective.

29. When I get an erection in the morning, it is stiff and rigid, but during sexual intercourse, it is not erect. It is better than before the treatment, but far from insertion.

30. I may not be satisfied with the result of the treatment because I fell ill after the treatment due to a lack of self-care.

31. I had great expectations after hearing the description, but it was a total disappointment.

32. I am glad I tried Morenova.

33. I enjoyed every treatment because I could listen to the doctor's valuable talks.

34. I will ask for Morenova again when the effect wears off after a while.

35. It was a little disappointing because the effect was not as good as I had expected. I feel the effect, but I also feel that the effect is fading as the months go by.

36. I would like to do a repeater campaign if there is one.

37. It has become difficult to ejaculate.

38. I am now able to get an erection even with a little stimulation, and I am very satisfied.

39. Thank you very much.

40. I am sorry that I am not able to practice until September due to various reasons, but I am in much better condition.

41. There were certain effects, but I don't feel that one treatment was sufficient.

42. For me, I could not feel much effect.

43. No particular change.

44. The progress is satisfactory.

45. I would do the treatment again if it were affordable.

46. I was starting to feel pretty old before I had it done, but it looked good enough.

47. I am wondering if it would be even better if I did it again.
